# Path planning and optimization for micro-robot in a vessel-mimic environment

**DOI:** 10.3389/fnbot.2022.923348

**Published:** 2022-09-07

**Authors:** Zhijie Huan, Weicheng Ma, Jiamin Wang, Feibin Wu

**Affiliations:** ^1^School of Electrical Engineering and Automation, Xiamen University of Technology, Xiamen, China; ^2^Quanzhou Institute of Equipment Manufacturing, Haixi Institutes of Research on the Structure of Matter, Chinese Academy of Sciences, Quanzhou, Fujian, China

**Keywords:** micro-robot movement, skeleton-extraction, A^*^ algorithm, safe distance analysis, path optimization

## Abstract

Manipulating micro-robots in blood vessels is an essential technology for medical researchers in applications such as drug delivery and thrombus removal. The usage of micro-robots in medicine can help overcome the limitations of many conventional clinical methods. In this study, we aimed to make the micro-robot more intelligent while moving through blood vessels. First, the skeleton of an image of the blood vessels is extracted, which is further used for path planning. Then, the skeleton-extraction-based A^*^ algorithm was used for determining a best route for the movement of the microrobot at a safe distance from the vascular wall. Finally, the gradient descent algorithm was utilized to smooth the planned path. Simulations were conducted to verify the effectiveness of the proposed algorithms. The proposed methods would improve the efficiency for the further manipulation of the micro-robot in the blood vessel environment.

## Introduction

In recent years, micro-robotics applications have been widely studied by biomedical engineering applications (Pan et al., 2009; Cheah et al., 2014; Bi et al., 2019; Abdelaziz and Habib, 2020; Jeon et al., 2020). In order to achieve accurate control of micro-robots, several control strategies have been explored; for example (Sabra et al., 2005), a 3D-path-planning algorithm for a microdevice through the blood vessels with MRI system. Meng et al. (2020) constructed a model-free reverse sliding mode controller for the navigation of micro-robots in the vascular environment and designed a smooth and efficient local planning trajectory. Xie et al. (2020) aimed to embed the biomimetic magnetic micro-robot with an internal magnetosome structure into non-expanding micro-solvents with accurate positioning control in order to enhance the thrombolytic effect on blood vessels. Park et al. (2010) proposed an integrated wireless-powered vascular micro-robot for the treatment of diseases, such as angina and cardiac infarction. Experiments were conducted for the investigation of motor performance, location recognition, therapeutic capacity, and overall control in blood vessels.

In order to increase the autonomous capability of micro-robots in complex vascular environments, real-time path planning could be introduced to improve the control performance. Conventional robot path planning operations used several different algorithms, such as the Dijkstra algorithm (Wang et al., 2011), A^*^ algorithm (Wang, 2021), RRT algorithm (Gong et al., 2014), ant colony algorithm (Wang et al., 2018), genetic algorithm (Liu et al., 2004), and artificial potential field algorithm (Qi et al., 2008). The A^*^ search algorithm is one of the most simple and efficient methods that have high adaptation and accuracy. However, in some situations, the conventional A^*^ algorithm (Qin et al., 2019) has several disadvantages, such as low search efficiency and a multiplicity of path turning points. Researchers have improved the heuristic function of the A^*^ algorithm (Shan and Meng, 2010) and combined a polynomial curve and a segmented polynomial function curve to smoothen the path. Path optimization with the secondary A^*^ search algorithm and a dynamic tangent point adjustment method on the basis of completing the global path planning has also been attempted (Wang et al., 2019). They also optimized the local path based on environmental information with the artificial potential field method to reduce the computation and improve the search efficiency. Those studies were mainly focused on the optimization of path smoothing and search efficiency of A^*^ algorithm. Nevertheless, in a complex vascular mimic environment, the micro-robot collides or develops friction with the vascular wall due to the deflection of motion during movement. Moreover, the movement trajectory of the micro-robot can also be influenced by environmental disturbance (Zhong et al., 2013, 2021; Lee et al., 2018; Guo et al., 2019). Therefore, we improved the path planning of the micro-robot on the basis of the A^*^ algorithm in order to guarantee a safe and smooth trajectory for the movement of micro-robots in blood vessels. The conventional A^*^ algorithm was combined with a map skeleton extraction method to obtain a safe route for the actual movement. Moreover, the smoothness of the route also has a significant effect on the control of the micro-robot in a vessel-mimic environment. The micro-robot experiences a more fluid movement with a relatively continuous speed. The gradient descent method (Chowdhury et al., 2000) was used for smoothening the movement trajectory.

## Methods

**Figure 1:**
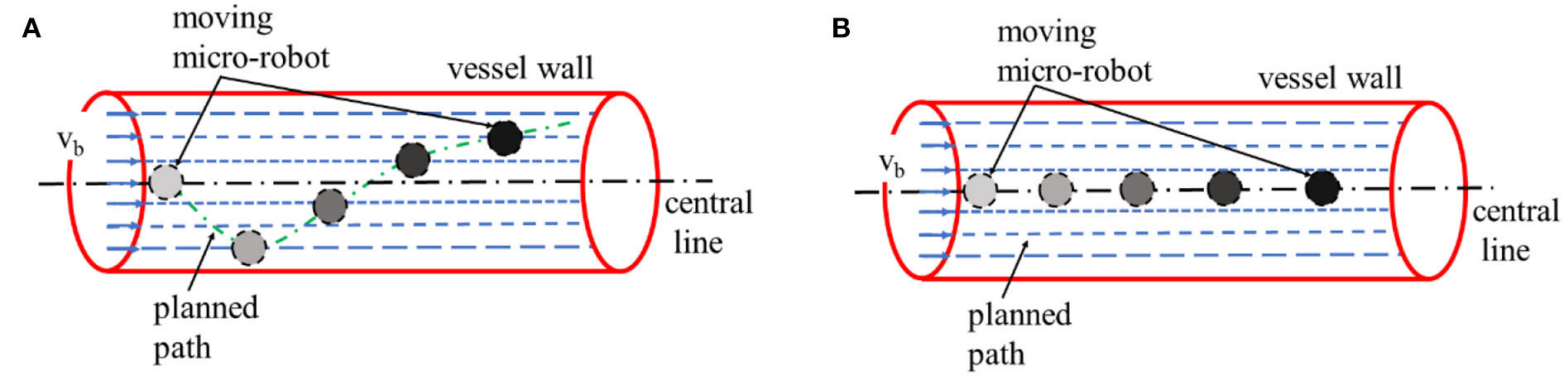
A schematic diagram for: **(A)** a micro-robot moving along a random route; **(B)** a micro-robot moving close to the central line of vessel.

Owing to the complexity of the vascular environment, the movement of the micro-robot is affected by the properties of the blood fluid (Liang et al., 2011). Based on the analysis of blood viscosity coefficient and blood flow velocity, it can be seen that the flowing blood may be divided into liquid layers with different flow rates under steady-state flow. The flow speed is the highest near the central line of the vessel, and is lowest close to the vessel wall. As shown in Figure 1A, if the micro-robot moves along a random route in the vessel, the blood speed varies according to the distance to the vessel wall, thus increasing the difficulty in control (Zhong et al., 2021). Because disturbances can also be induced by the different microfluidic environments, the micro-robot can deviate easily from the desired route, and scratch or even collide with the blood vessel wall, which would cause unpredictabilities in the motion state. Therefore, we tried to set a safe and stable path for the micro-robot away from the vessel wall along the central line of the microfluidic environment, where the blood has steady flow velocity, as shown in Figure 1B (Zhang, 2008; Qi and Xu, 2010; Biswas et al., 2014; Shi and Long, 2014; Deng and Huang, 2017; Jiang et al., 2020). The skeleton of the vessel-mimic map was extracted, with which we could obtain the center line of the passable area through refinement iteration.

The vessel-mimic map was imported and reconstructed with binary processing. Then, the morphological operation method of corrosion and expansion was used to achieve boundary elimination and burr removal. After the image was open processed, the center line position of the map was obtained by refining the iterative over-extracted skeleton. A skeleton channel with the width of only one pixel was extracted without changing the image boundary. Finally, modified A^*^ algorithm was used to search within the skeleton channel. This method could make sure that the microrobot was at a safe distance away from the vessel wall, which could also enhance the search efficiency for path planning.

In order to further test the proposed algorithm, two maps mimicking the vessel environment were constructed. To enable the navigation of the microrobot along the central skeleton of the map, the grid size of the map was set to be 20 μm × 20 μm, matching the size of the microrobot during preprocessing. Skeleton extraction was performed with the two proposed maps, respectively.

## Results and discussion

### Path planning simulation

**Figure 2:**
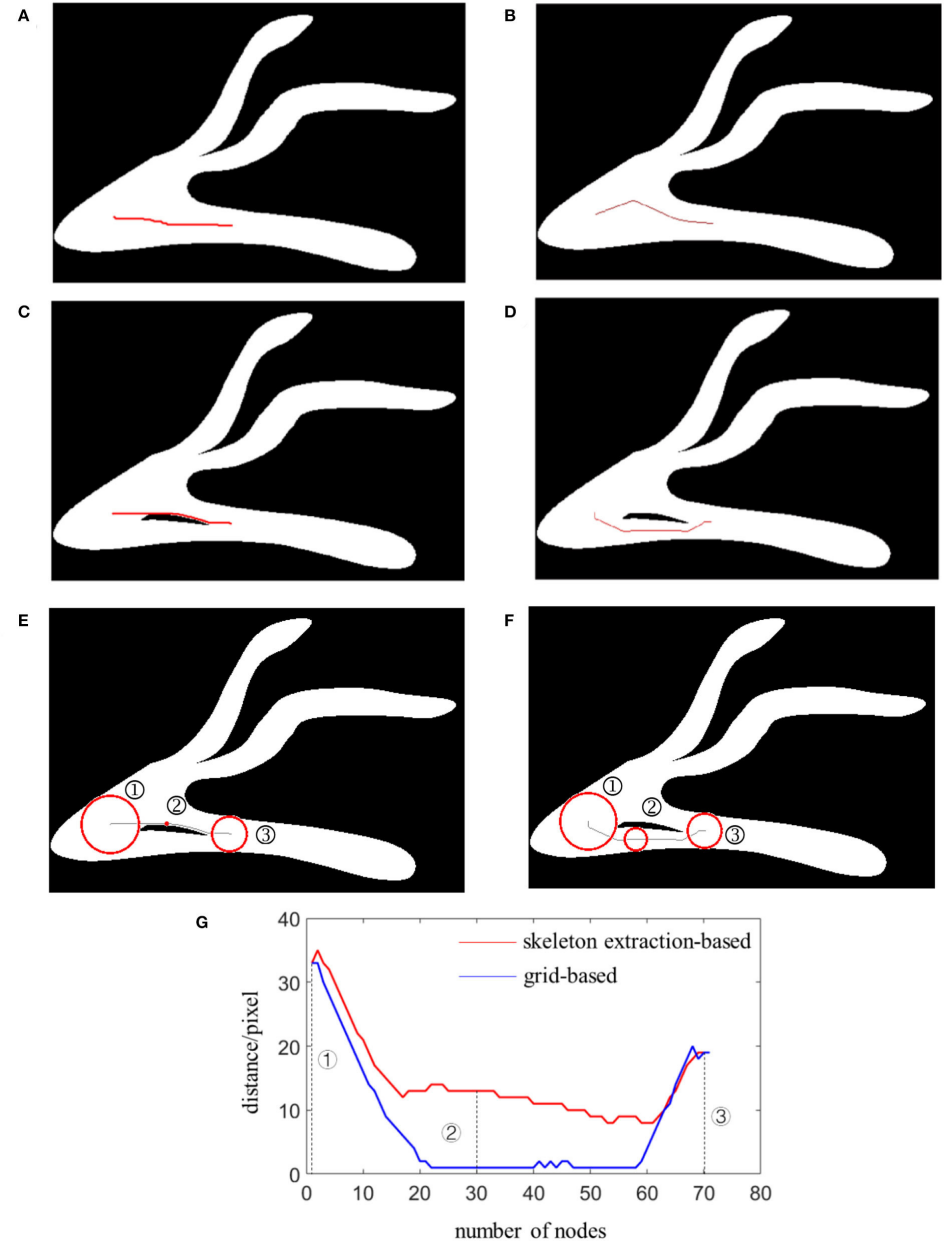
Path planning in Map 1 with: **(A)** grid-based A*algorithm; **(B)** skeleton-extraction-based A*algorithm; path planning in Map 2 with: **(C)** grid-based A*algorithm; **(D)** skeleton-extraction-based A*algorithm; distance evaluation for Map 2 planned with **(E)** grid-based A*algorithm; **(F)** skeleton-extraction-based A*algorithm; **(G)** an analysis curve graph.

As mentioned in section Methods, it is necessary to ensure that the movement of the micro-robot in the blood vessel does not collide or scratch with the vessel wall. Pretreatment for the environmental map by extracting the skeleton can solve the problem, which can keep the micro-robot at a safe distance from the blood vessel wall and avoid obstacles during the movement. After the extraction of the map skeleton, these nodes were retained for further navigation. The A^*^ algorithm was used to find a feasible path from the start point to the end. The heuristic function was utilized to estimate the cost distance between the neighboring nodes, find the series of minimum nodes, and search an optimal path for the micro-robot. As shown in Figure 2, simulation experiments were conducted with the constructed vessel-mimic environment maps. The path search procedure was performed with the A^*^ algorithm in the original maps and in the skeleton-extracted maps, respectively. The simulation results in Figures 2A,B illustrate no significant difference between the planned routes with the A^*^ algorithm in a grid-based map and a skeleton-extracted map. This was mainly because there are no obvious obstacles within the given Map 1. However, in an actual vascular environment, some abnormal tissue structures may be present as can be observed from Map 2, given in Figures 2C,D, which influences the normal movement path for the micro-robot.

The skeleton is extracted to preprocess the map and obtain a safe path for the micro-robot moving in blood vessels to avoid collision and friction with the vessel wall. As shown in Figure 2C, with the furcation between the start point and the target point, the planned path will guide the micro-robot moving close to the vessel wall. However, with pretreatment *via* the skeleton extraction, Map 2 was reduced to several simple lines. On this basis, the points in the planned path always keep a maximum distance from the vessel wall, as illustrated in Figure 2D.

### Efficiency analysis

The simulation results in Figure 2C show that the path planned for the micro-robot movement based on the grid-based A^*^ algorithm in Map 2 cannot maintain a safe distance away from the obstacle. For some nodes, the micro-robot moves almost close to the edge of blood vessels. With such a short distance, the possibility of collision between the micro-robot and the vascular wall increases. As illustrated in Figures 2E,F, the distance between the micro-robot and the vascular wall was presented in three typical nodes for the path planned with the grid-based A^*^ algorithm and skeleton-extraction-based A^*^ algorithm. The radiuses of the circles in the red line represent the minimum distance from the vascular wall. For the first node and the third node, there is no significant difference between the two methods. However, for the second node, a suitable distance could be ensured using the path planned with the skeleton-extraction-based A^*^ algorithm.

The entire path consists of 70 nodes as shown in Figure 2G. The path planned with the skeleton-extraction-based A^*^ algorithm always maintained a relatively safe distance from the edge of the blood vessel given in the red curve. That is, the micro-robot could move in the center of the blood vessel, avoiding the possibility of collision and changing of flow velocity. However, for the path planned with the grid-based A^*^ algorithm illustrated in blue, most of the nodes are close to the edge <5 pixels. Accuracy control for the micro-robot was extremely difficult with the increased disturbance.

To further evaluate the efficiency of the two proposed algorithms, the planning time required for the entire process was quantitatively analyzed. As presented in Table 1, most of the time for skeleton-extraction-based A^*^ algorithm was spent in map preprocessing. Since the skeleton extraction in the improved A^*^ algorithm dramatically reduced the range of target nodes, it is less time-consuming compared with the grid-based A^*^ algorithm.

**Table 1:** Path planning efficiency.

**Environment**	**Algorithm**	**Preprocessing time (s)**	**Total processing time (s)**
Map 1	Grid-based A*	0	93.43
	Skeleton-extraction-based A*	1.61	1.63
Map 2	Grid-based A*	0	107.70
	Skeleton-extraction-based A*	1.62	1.62

### Path optimization

In addition, to ensure a safe distance within the planned route, the smoothness of the route also has a significant effect on the control of the micro-robot in a vessel-mimic environment. The micro-robot experiences a more fluid movement with a relatively continuous speed. Therefore, we utilized the image skeleton-extraction method to make up for the shortcomings of the grid-based A^*^ algorithm. The original path was limited to the extracted single pixel channel. With the obtained discrete path points, the gradient descent method was further introduced to smooth the path.

The gradient descent method is a kind of algorithm that can solve a minimum value for the target function, which is commonly used to fix unconstrained optimization problems. In the path smoothing process, the main objective is to find a series of minimum distance, which stands for the offset of the path point sequence before and after the transformation. Assume that the original planned path point sequence in the environment is *X*(*n*), and the smooth path point sequence is *Y*(*n*). The route smoothing process serves to obtain the minimum values of Equations (1) and (2), respectively. With multiple iterations, the minimum value for the objective function in Equation (3) could be acquired.


(1) $$\begin{array}{lll} D\left(n\right) & = & {\left(∥X\left(n\right)-Y\left(n\right)∥\right)}^{2} \end{array}$$

(2) $$\begin{array}{lll} D_{y}\left(n\right) & = & \left(∥Y\left(n\right)-Y\left(n+1\right)∥\right) \end{array}$$

(3) $$\begin{array}{lll} Y_{min} & = & {\alpha \;*\;∥X\left(n\right)-Y\left(n\right)∥}^{2}+\beta \;*\;∥Y\left(n\right)-Y\left(n+1\right)∥ \end{array}$$

where α is the length of the learning step, and β is the degree of smoothness. D (n) is the degree of deviation between original points and optimized points. D_y_(n) is the distance between the adjacent two points after optimization. The effect of smoothness for the target route varies as the values of the parameters change. As shown in Table 2, the results of the path-smoothing simulation results of different parameters were listed. In order to facilitate observation, the original planned path point sequence in the presented red line and the blue line stands for the smoothed route.

**Table 2:** Path smoothing simulation results with different parameters.

**α, β**	**Maximum rate of slope variation**	**Maximum offset distance**	**Path smoothing effect**
α = 0.9 β = 0.1	75.7	0.0014	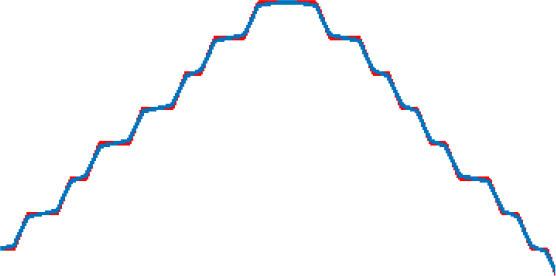
α = 0.7 β = 0.3	46.7	0.0239	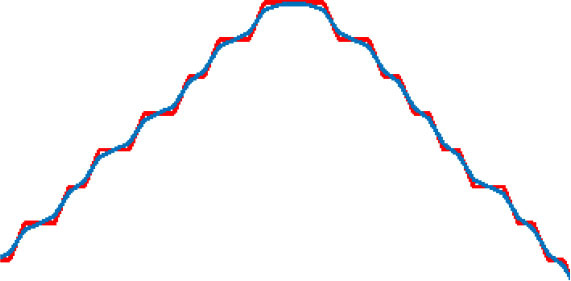
α = 0.5 β = 0.5	30.1	0.0859	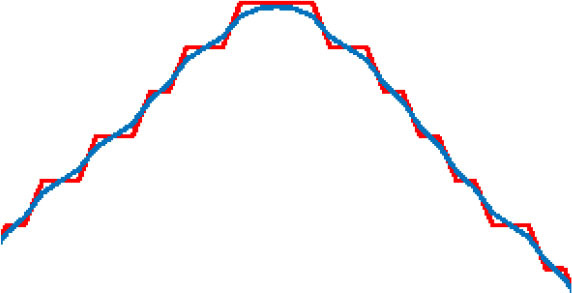
α = 0.3 β = 0.7	19.1	0.2275	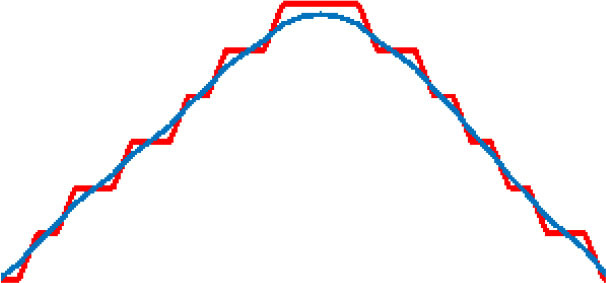
α = 0.1 β = 0.9	10.0	0.7366	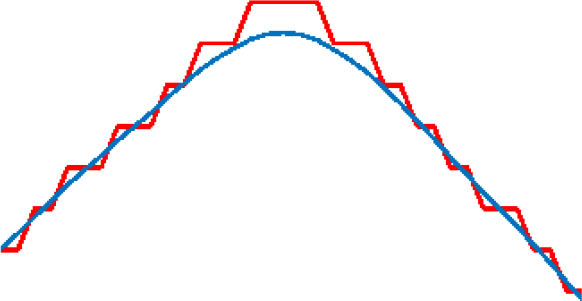

After the simulation, the maximum rate of slope variation and the maximum offset distance for each optimized path were analyzed for further evaluation of the smoothening effect. The results indicate that, with different α and β inputs, the smoothness of the optimized path varies obviously. In order to ensure a safe path for the micro-robot movement, a minimum offset distance should be satisfied when choosing the parameters. Through comparison of the original points and the optimized curve points, the offset of the smoothed path moves up and down with the change in the parameter value. Thus, we analyzed the average variance around the inflection point of the optimized curve with different parameters. The appropriate parameters could be obtained, considering both the smoothness effect and the offset distance.

Five different groups of parameters were introduced for the simulation experiments. The results are listed in Table 2, respectively. According to the distribution of the result data, the smaller the learning step and the larger degree of smoothing, the smoother the processed curve obtained. However, the maximum offset distances of the processed curve increased accordingly. On the contrary, the smoothness of the processed curve deteriorates. In order to achieve the minimum deviation distance and a better smoothing effect concurrently, the maximum rate of slope variation was taken into consideration. With the smaller difference between each of the two adjacent points after the smoothing process, the smoothness of the curve improved. In contrast, a large rate of slope change indicates more mutation after the smoothing process, and the smoothing effect is relatively worse. Because the micro-robot should move along a safe path, which is based on the skeleton extraction, the maximum offset of the optimized path cannot exceed the 50% width of the skeleton channel. The data analysis in Table 2 shows that the smallest maximum offset distance is with the parameter of α = 0.9 and β = 0.1. However, the slope variation in this situation was the largest, which contains lots of inflection points. The best smoothness is obtained when α = 0.1 and β = 0.9, but the maximum offset distance of the path is the largest. When α = 0.5 and β = 0.5, the curve is relatively well-smoothed, and the maximum offset distance is within a reasonable range. Therefore, considering the requirement of the safe distance and smoothness of the optimized path, the processing parameter is selected as α = 0.5 and β = 0.5.

## Conclusion

This study introduces the A^*^ path-finding algorithm based on skeleton extraction to ensure the safety of micro-robot movement in the vascular environment, extracts the environment skeleton, and uses the A^*^ search to obtain single-pixel-channel-path trajectories. By fitting the circle, the resulting path becomes safe again. The algorithm is greatly shortened, and the road-finding speed is rapidly improved. The gradient descent method is adopted to smoothen the path, which finally results in a safe and smooth path of the microrobot in the vascular environment.

## Data availability statement

The original contributions presented in the study are included in the article/supplementary material, further inquiries can be directed to the corresponding author.

## Author contributions

ZH and WM: conceptualization, methodology, validation, data analysis, and writing–original draft preparation. ZH and JW: materials development, writing, review, and editing. All the authors have read and agreed to the published version of the manuscript.

## Funding

This work was supported by the National Natural Science Foundation of China (NSFC) under grant Nos. 61903315 and 62003285, and the Natural Science Foundation of Fujian Province under grant Nos. 2019J05124, 2019J01869, 2020J02045, and 2020J01285.

## Conflict of interest

The authors declare that the research was conducted in the absence of any commercial or financial relationships that could be construed as a potential conflict of interest.

## Publisher's note

All claims expressed in this article are solely those of the authors and do not necessarily represent those of their affiliated organizations, or those of the publisher, the editors and the reviewers. Any product that may be evaluated in this article, or claim that may be made by its manufacturer, is not guaranteed or endorsed by the publisher.
